# Supplementary material for: A binning tool to reconstruct viral haplotypes from assembled contigs
Source: BMC Bioinformatics. 2019 Nov 4;20:544. doi: 10.1186/s12859-019-3138-1 (PMC6829986; doi:10.1186/s12859-019-3138-1)
Supplement: Supplementary file 1 — Additional file 1 aforementioned experimental results. [file 12859_2019_3138_MOESM1_ESM.pdf]

Additional file 1

1. Data simulation details

Fig. S1 sketches the input data sets for the simulated contigs.

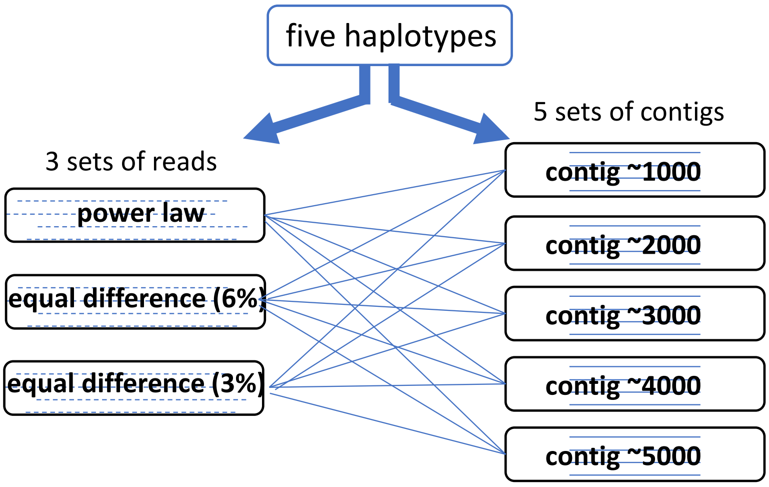

Fig. S1. The construction of 15 input contig sets. The five contig sets are randomly generated from the haplotypes with different length distributions.

The details about the contig sets can be found in Table S1. ``Len (longest)'' is the length of the longest contig in a group. ``Genome coverage'' is the percentage of the underlying genomes covered by all the simulated contigs. ``Group ID'' (1000 to 5000) indicates the upper bound of the contig length in each group.

Table S1. Properties of simulated contains for five haplotypes.

Group ID	1000	2000	3000	4000	5000
contaig number	61	35	30	25	24
Len(longest)	1000	1956	2748	3940	4879
N50	786	1568	1993	2518	2525
Genome converage (%)	88.7	91.3	91.4	93.6	96.6

2. Abundance prediction by VirBin on the assembled contigs

The contigs are produced by assembly tools SGA and PEHaplo on simulated reads from five haplotypes. The details of the assembled contigs can be found in Table S2

Table S2. Assembly results on three simulated read sets with PEHaplo and SGA.

Data set	Tools	Contigs number	N50	Genome cov. (%)	Longest contig	Mismatch rate (%)
Power	PEhaplo	6	9513	97.4	9727	0.002
	SGA	17	4963	99.4	9727	0.002
Equal-6%	PEhaplo	6	9667	97.9	9753	0.004
	SGA	16	5276	99.2	9667	0.004
Equal-3%	PEhaplo	18	3040	98.0	9463	0.0
	SGA	17	3385	99.6	9684	0.0

N50 is defined as the maximum length in which all contigs of at least this length contain at least 50% of all the contig bases. Genome coverage (cov.) is the percentage of reference genomes that are aligned by contigs, and mismatch rate is the percentage of mismatches of aligned contigs.

The relative abundance is computed during the iterative clustering algorithm in VirBin. Fig. S2 compares the known haplotype abundances with computed ones by using assembled contigs as input for VirBin. The abundance profiles output by VirBin on both PEHaplo's and SGA's assembled contigs are close to the known haplotype abundance profile. As there are many empty clusters for MaxBin's results, we did not include the abundance comparison.

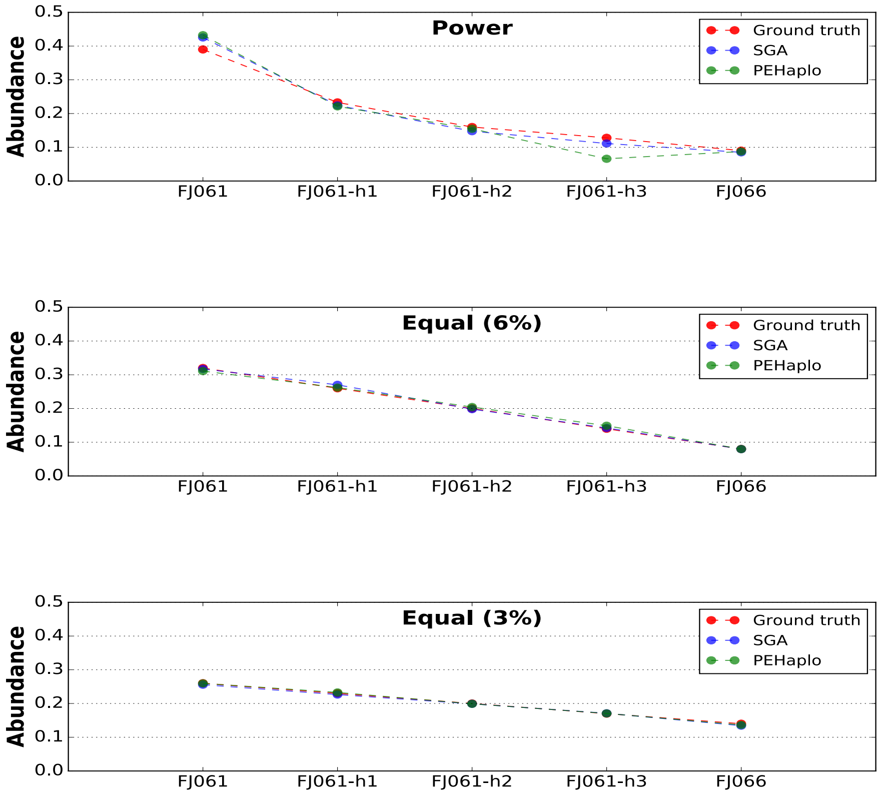

Fig. S2. The comparison between the ground truth abundance distribution and predicted abundance distributions by applying VirBin on assembled contigs by PEHaplo and SGA.

3. Results for 10 HIV haplotypes

The simulated 10-haplotype quasispecies contain FJ064, FJ061, FJ065, FJ066, and two simulated haplotypes from the last three strains. The sequence similarity between the simulated haplotype and its originating sequence is 97%. The average sequence similarity between all the 10 haplotypes is around 90.1%. In total, there are 76,974 reads with sequencing depth 2000-x. The relative abundances for each haplotype are shown in Fig.S3. Using the same simulation method as the 5 HIV haplotype data set, 25 contigs were generated, covering 89.45% of the 10 haplotypes. The longest contig has length 8846bp and the N50 is 5933.

Haplotype number estimation: The contig alignment and windows identification were also applied on simulated contigs. We sorted the windows in descending order of window length. Out of the top 50 windows, 26 have 10 contigs, 16 contain 9 contigs, and 6 contain 8 contigs.Therefore, the haplotype number 10 can still be correctly predicted by the consensus window depth.

Clustering results: The clustering results by VirBin are shown in Table S3. The 3 least abundant haplotypes have highly similar abundances (~ 3%) and got the lowest recall and precision values. The results were also compared with MaxBin. 10 seed contigs from each haplotype were randomly selected and provided to MaxBin. It classified 25 out of 34 contigs, with 9 unclassified. MaxBin correctly classified all contigs from FJ064 and FJ061-h1, and one contig from FJ061-h2. It assigned three contigs to FJ066 cluster with one being correct. Most of the other contigs were not appropriately clustered. The results of MaxBin are shown in Table S3 as well. We again tried StrainPhlAn and ConStrain on this simulated data set, but still, no reads can be mapped to available reference genes.

Table S3: Clustering results on simulated 10 HIV haplotype contigs for VirBin and MaxBin. The haplotype is sorted in descending order to relative abundance.

	VirBin		MaxBin	
	Precision(%)	Recall(%)	Precision(%)	Recall(%)
FJ061	100.0	80.0	0.0	0.0
FJ066	84.6	95.8	43.4	62.4
FJ065	86.4	61.5	0.0	0.0
FJ064	73.0	98.0	18.8	100.0
FJ061-h1	98.1	88.1	44.7	100.0
FJ061-h2	99.5	89.2	100.0	26.6
FJ066-h1	90.0	79.2	0.0	0.0
FJ066-h2	60.0	47.6	0.0	0.0

	VirBin		MaxBin	
	Precision(%)	Recall(%)	Precision(%)	Recall(%)
FJ065-h1	18.6	10.7	0.0	0.0
FJ065-h2	42.1	94.9	0.0	0.0

Fig. S3 compares the true abundance distribution with the output of VirBin and MaxBin. It is not hard to see that VirBin's output is closer to the ground-truth than MaxBin.

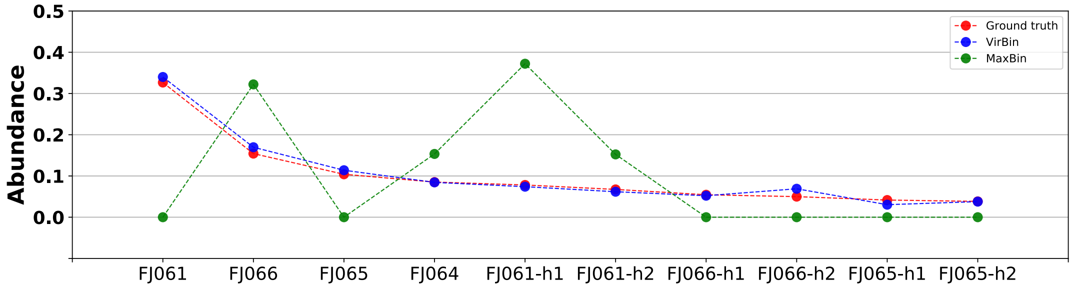

Fig. S3: The relative abundance distribution for 10 haplotypes and the output relative abundance profile by VirBin and MaxBin.

4. Additional results for the mock data set

For the mock data experiment, we also present the recall and precision at contig level in Table S4. Thus, the recall quantifies how many of the contigs originating from one haplotype are correctly grouped in one cluster. The precision quantifies how many contigs in a cluster originate from the corresponding haplotype. Our program is still generally more accurate than MaxBin.

Table S4: Contig-level clustering results on assembled 5 real haplotype contigs for VirBin and MaxBin. The recall and precision are evaluated on contigs rather than bases. The haplotypes are sorted in decreasing order of abundance.

	VirBin		MaxBin	
	Precision(%)	Recall(%)	Precision(%)	Recall(%)
JRCSF	33.3	33.3	0.0	0.0
NL43	66.7	28.6	25.0	20.0
89.6	33.3	100.0	0.0	0.0
YU2	25.0	20.0	33.3	33.3
HXB2	70.0	87.5	33.3	25.0
